# Comparison of Sequencing Based CNV Discovery Methods Using Monozygotic Twin Quartets

**DOI:** 10.1371/journal.pone.0122287

**Published:** 2015-03-26

**Authors:** Marc-André Legault, Simon Girard, Louis-Philippe Lemieux Perreault, Guy A. Rouleau, Marie-Pierre Dubé

**Affiliations:** 1 Faculty of Medicine, Université de Montréal, Montreal, Quebec, Canada; 2 Department of Human Genetics, McGill University, Montreal, Quebec, Canada; 3 Beaulieu-Saucier Pharmacogenomics Center, Montreal Heart, Institute Research Center, Montreal, Quebec, Canada; 4 Montreal Neurological Institute and Hospital, McGill University, Montreal, Quebec, Canada; Institut Jacques Monod, FRANCE

## Abstract

**Background:**

The advent of high throughput sequencing methods breeds an important amount of technical challenges. Among those is the one raised by the discovery of copy-number variations (CNVs) using whole-genome sequencing data. CNVs are genomic structural variations defined as a variation in the number of copies of a large genomic fragment, usually more than one kilobase. Here, we aim to compare different CNV calling methods in order to assess their ability to consistently identify CNVs by comparison of the calls in 9 quartets of identical twin pairs. The use of monozygotic twins provides a means of estimating the error rate of each algorithm by observing CNVs that are inconsistently called when considering the rules of Mendelian inheritance and the assumption of an identical genome between twins. The similarity between the calls from the different tools and the advantage of combining call sets were also considered.

**Results:**

ERDS and CNVnator obtained the best performance when considering the inherited CNV rate with a mean of 0.74 and 0.70, respectively. Venn diagrams were generated to show the agreement between the different algorithms, before and after filtering out familial inconsistencies. This filtering revealed a high number of false positives for CNVer and Breakdancer. A low overall agreement between the methods suggested a high complementarity of the different tools when calling CNVs. The breakpoint sensitivity analysis indicated that CNVnator and ERDS achieved better resolution of CNV borders than the other tools. The highest inherited CNV rate was achieved through the intersection of these two tools (81%).

**Conclusions:**

This study showed that ERDS and CNVnator provide good performance on whole genome sequencing data with respect to CNV consistency across families, CNV breakpoint resolution and CNV call specificity. The intersection of the calls from the two tools would be valuable for CNV genotyping pipelines.

## Introduction

Copy Number Variants (CNVs) are large (>1 kilobase) genomic structural variations [1, 2]. The phenotypic effect of such changes in genomic structure can result in perturbations in gene dosage, gene interruption, gene fusion or the unmasking of recessive alleles [3]. The implication of CNVs in the etiology of human diseases has been previously established in the case of both Mendelian and complex disorders [4, 5]. These important biological consequences motivate the refinement of the methods used to detect CNVs using modern genetic datasets such as whole genome next-generation sequencing data. The current state of the detection methods has limited the widespread study of CNVs in the context of phenotypic associations, in part because of the limited characterization of their genomic structure. The development of bioinformatics protocols and tools producing high confidence, consistent CNV calls will be the first step leading to the thorough investigation of such associations. Accurate characterizations of CNV calling tools have been published in the context of whole exome sequencing [6]. This technology being different from whole genome sequencing in terms of systematic biases (*e*.*g*. due to exome capture) and genome coverage, the underlying algorithms are often different than for whole genome sequencing. Recent method comparisons have also addressed tools suited for whole genome data, but often restricted their scope to an algorithmic family such as *depth of coverage* methods [7]. Here, we will compare methods from all the different commonly used algorithmic methods for CNV discovery in whole genome sequencing data. The aim of integrating different methods in this comparison is to derive an optimal CNV call set regardless of the underlying bioinformatics strategies.

One of the first methods used for CNV detection was comparative genomic hybridization arrays (aCGH). This technique co-hybridizes differently labelled genomic DNA from ‘test’ and ‘reference’ cell populations to an array of probes and compares the ratio of fluorescence to detect significant variation in copy-number [8]. Unfortunately, the probe density and the size of the targets limit the resolution of the CNV breakpoints. Estimates of the resolution of aCGH vary between 0.25Mb and 2Mb depending on the difference in copy number [9, 10]. Another method for CNV detection uses genotyping arrays. These methods rely on the use of single nucleotide polymorphisms (SNP) arrays to assess continuous genomic regions of low or high probe intensity representing regions of decreased or increased copy number, respectively. Hence, the precision of the detection is dependent on the distribution of the probes in the genome, as regions of high probe density will be more thoroughly assayed. Those experimental biases make the confident discovery of new CNVs and the precise resolution of their breakpoints harder.

Modern techniques, including whole genome high-throughput sequencing, partially overcome these disadvantages by providing increased breakpoint resolution, robust copy number estimation and the ability to discover structural variants using paired-end sequencing signatures and depth of coverage. The interpretation of such data still requires algorithmic developments in order to increase sensitivity and specificity. Current methods can be divided in four categories: (1) read depth, (2) paired-end mapping, (3) split-reads and (4) assembly-based methods. Read depth methods detect CNVs according to the number of sequenced reads mapped to a given genomic position. This concept is analogous to the SNP microarrays where the fluorescence intensity at a given probe allows the detection of similar events [11]. Paired-end mapping methods use information from the distance and orientation between sequencing reads issued from the same insert to detect structural variants (including, but not limited to CNVs) [12]. Split-reads methods use paired-end reads where one of the reads is mapped to a given genomic region and the other pair fails to map, which helps define the breakpoints of CNVs [13]. Assembly-based methods rely on the *de novo* assembly of sequences to discover insertions and deletions that are absent from the reference [14]. The short read length currently available from next-generation sequencing, however, makes this approach impractical in the case of large, complex genomes such as the human genome [15]. Some hybrid methods also combine different approaches in order to broaden the spectrum of information used in CNV discovery. It has been discussed that the combination of different information types may help achieve better CNV calls [16].

In the present study, 9 quartets composed of monozygotic twins and their parents have been sequenced using whole-genome sequencing. Different CNV discovery tools have been used on every sample allowing a thorough comparison of the selected methods by comparing the calls from both twins. The use of monozygotic twins allows the assumption of an identical genome when ignoring *de novo* CNVs since they account for a very small portion of the total CNV burden. Tracking variants transmission from parents to their offspring provides a useful metric (rate of inherited CNVs) to compare the tested algorithms.

## Methods

### CNV calling tools

Four CNV-calling tools have been selected for comparison (CNVer, BreakDancer, CNVnator and ERDS) (Table 1). The selection of these tools was based on the differences in their underlying algorithmic approaches. At least one algorithm of each of the main categories was selected (paired-end mapping, read depth and hybrid). Assembly based methods were not used as they are dependent on the capacity of deriving confident genome assemblies from the short reads issued from high throughtput sequencing platforms. The algorithm's efficiency were assessed by using whole-genome sequencing data from 9 monozygotic twin quartets.

**Table 1:** Summary of the selected CNV calling algorithms.

Algorithm	Category	Summary characteristics	Reference
CNVer	Hybrid (PEM + RD)	Builds a donor graph integrating PEM and RD information. Requires specific mapping with Bowtie.	[12]
BreakDancer	PEM	Detects CNVs, inversions and translocations. Provides Phred-like score. Software for small indels (10–100 bp) also available.	[17]
CNVnator	RD	Uses a mean-shift technique to partition genomic bins in segments of different copy number.	[18]
ERDS	Hybrid (PEM + RD + SC*)	Uses a HMM to combine SNV heterozygosity and RD information. Supports calls with PEM and SC signatures. Requires a Variant Call Format file and high coverage (> 20X is recommended)	[19]

*Soft-clipping signatures are analogous to split-reads signatures

PEM stands for Paired-End Mapping, RD for Read Depth and SC for Soft Clipping.

**CNVer (v0.8.1)** uses information from both paired end mapping and read depth methods in a graph-theoretic framework to call CNVs. The paired-end mapping make it possible to create linking clusters of discordant read pairs which span the breakpoints of CNVs. The read depth signatures are then combined to the previously described information in a data structure called the donor graph, representing the donor genome in a compact manner. The raw output from CNVer is a file identifying every genomic region of “gain” or “loss” with its corresponding depth of coverage ratio (computed in small genomic windows to avoid bias from GC content). A separate tool is provided to infer the absolute copy number counts after CNV calling [12]. To call CNVs with CNVer on our dataset, we defined the read length as 100 bp, the computed mean insert size was of 314 bp (σ = 63) and at least 20 mate pairs were required to form a cluster, which is reasonable given the coverage in our dataset. Based on a paired-end mapping method, **BreakDancer (v1.1.2)** classifies structural variations in six different categories: normal, deletion, insertion, inversion, intrachromosomal translocation and interchromosomal translocation. Genomic regions with a statistically significant amount of anomalous read pairs are then considered to be structural variations breakpoints. A *Poisson* model considering the number of supporting anomalous reads, the size of the region and the genomic coverage is used to compute a confidence score that is converted to a Phred scale score ($Q\;=\;-\log_{10}P$ where Q is the Phred scale score and P is a *p*-value). BreakDancer’s true positive rate (TPR) is correlated with the physical coverage. According to simulated data, a 30X fold physical coverage corresponds to a 20% TPR [17]. The default parameter values were used for BreakDancer.

**CNVnator (v0.2.7)** uses read depth information in non-overlapping genomic bins of identical size (user defined) to identify segments of different copy number, which represent putative CNV breakpoints. The mean-shift technique is used to identify such segments, and their statistical significance is assessed using a *t*-test statistic (p<0.05) corrected for multiple hypothesis testing. The scenario where large CNVs would be fragmented in multiple smaller calls has been resolved by merging adjacent calls if the read depth signal is identical in both calls and the region in between [18]. We used a bin size of 1,000 for the analysis presented in this paper. Such a large bin size should lead to a higher confidence calls, but weaker breakpoint resolution.

**ERDS (v1.1)** first partitions the genome into regions deemed amplified and non-amplified by considering the ability of the aligner to distinguish them. This strategy is meant to avoid calling CNVs independently in paralogous loci. Similarly to other read depth methods, a sliding, fixed-size genomic window is used to compute the read depth across the genome. For the non-amplified regions, a hidden Markov model (HMM) is then used to combine single nucleotide variation (SNV) heterozygosity and read depth data in order to detect CNVs. Additional information from paired-end mapping and soft-clipping signatures is then combined with the previously established calls to either reinforce low-confidence calls or to detect CNVs that were undetected by the HMM [19]. Soft-clipping signatures are similar to split-reads signatures as they rely on the parts of a read that are not mapped to the reference (soft-clipped subsequences) to detect SV breakpoints [20]. ERDS does not provide hyperparameters to tune the bahaviour of the underlying algorithm.

### Sequencing

DNA from the 36 individuals was extracted directly from blood provided by the Quebec Study of Newborn Twins [21]. Libraries were constructed according to Illumina standard protocols. Sequencing was done on an Illumina HiSeq 2500 at Illumina sequencing facilities. Paired-end mode was used and the median fragment length was found to be ~300 bp for every individuals. The raw sequence data has been submitted to the European Nucleotide Archive (ENA). The study accession number is PRJEB8308.

### Mapping and variant calling

The paired-end reads were mapped to the reference genome (GRCh37) using the Burrows-Wheeler Aligner (bwa version 0.6.2-r126-tpx), increasing the quality threshold for read trimming from 0 to 5 [22]. The Genome Analysis Toolkit (GATK v1.6) [23] was used for indel realignement, read duplicate removal and quality score recalibration. The GATK DepthOfCoverage tool was used to generate quality control metrics. The mean depth of coverage was 36.9 (σ = 3.4). Indels and SNVs were called using GATK UnifiedGenotyper, because ERDS relies on the generated VCF files for CNV genotyping. For CNVer, a specific alignment was strongly recommended by the authors. Therefore, the reads were mapped to the reference using the Bowtie tool (v0.12.9) [24] with options restricting the reads to the single position with the least number of mismatches (options “-v 3-a-m 600—best—strata”).

### Analysis

In order to have comparable calls between the different tools, only the CNVs were kept, even if a given algorithm had called other structural variations. The numeric copy number was not considered for the analysis, only the “gain” or “loss” status was used for a copy-number above or below 2, respectively. Distinct calls having the same status and separated by less than 5 kb were merged in order to remove biases resulting from call fragmentation, which can occur if no statistical testing is used to insure the region between adjacent calls is not part of the same biological entity. Except for CNVer, this merging procedure had little effect on the calls, but more than 50% of CNVer's calls were affected, suggesting a high call fragmentation for this algorithm (S1 Fig.). All other analyses were conducted using the Python programming language and the set of tools (CNV Analysis Toolkit) available on StatGen’s website (http://www.statgen.org). Documentation and source control can be found on StatGen’s Bitbucket page (https://bitbucket.org/mhi_statgen/cnvanalysistoolkit). For two CNVs to be considered "equal", the concept of reciprocal overlap (RO) threshold was used. Here, we define RO as a tuple of the number of overlapping bases divided by the size of both of the compared CNVs. Unless otherwise specified, a threshold of 70% was used for all the analysis, meaning that when comparing two copy number variable regions, the number of overlapping bases divided by the whole region had to be more than 70%. The inherited CNV rate is defined as the number of CNVs shared by both twins and at least one parent divided by the total number of CNVs called for the twins. Concretely, it was computed as $2\times shared/(N_{1}+N_{2})$, where *shared* corresponds to the previously described CNVs and *N* are cardinalities of the twin’s CNV call sets.

The familial relationship classification test was used to estimate the randomness in CNV calls under the assumption that if the calls are specific, unrelated and genetically related individuals would be easy to distinguish. The 9 pairs of twins were used along with the corresponding 18 pairs of child-parent and 9 randomly generated pairs of unrelated individuals. The rate of shared CNVs in each pair was then computed (S2 Fig.) and the k-means algorithm was used in order to create three clusters representing the familial relationships. The classification corresponding to the previously described clustering was used to compute the F1-Scores for every tool. Concretely, this score corresponds to the weighted average of precision and recall: $F_{1}\;=\;2∙(P∙R)/(P+R)$ where P is the precision and R is the recall. This metric is often used to evaluate classification test accuracy and, in this case, provides insight over the existence of systematic bias of the genotyping method. For both the k-means and the F1-Score computations, the implementation from scikit-learn was used [25].

### Ethics statement

The patients gave informed written consent and the ethics committee of the CHU Sainte-Justine approved the project.

## Results and Discussion

### CNV call characterization

In order to evaluate the characteristics of the CNV calls obtained from the tested algorithms, we computed statistics serving as assessment metrics, which included the number of calls per sample, the size of the calls, the mean distance between adjacent calls and the total portion of the genome covered by CNV calls. Table 2 summarizes the results of these statistics for the different tools after merging the CNVs as described in the Methods. These statistics help characterize the systematic behaviour of the algorithms such as CNV size overestimation, call fragmentation (where a single true CNV is fragmented into multiple calls) or a high false positive rate. It is noticeable that the mean number of CNVs genotyped by Breakdancer is considerably larger than what is expected when comparing to the other tools. One could argue that this high number of calls, which are largely separated by a small number of bases (4kb), is symptomatic of a higher false positive rate. It is also interesting to note that the CNV size is smaller, on average, for Breakdancer and larger for CNVnator. This could be attributable to the type of information used by the underlying algorithms (paired-end mappings versus read depth). CNV size overestimation by read depth methods due to poor breakpoint resolution has previously been observed [15, 16] and justifies the high nucleotide based genome coverage of CNVnator. Larger studies also estimate the genome coverage of CNVs to be between 3.7% and 12%, which is higher than what all tools except CNVnator detected in our study [26–28]. This can be attributed the tested tools’ inability to detect all of the CNVs in a given genome. It could also be due to an overestimation of the genome coverage because of systematic CNV size overestimation in the literature [29].

**Table 2:** Characterization of the CNV calls for the different tools.

	Mean Number of CNVs	Mean size (kilobase)	Mean distance between adjacent CNVs (kb)	Genome coverage	Inherited CNV rate	Familial classification F1 Score
CNVer	1751 (72)	38 (2)	1259 (77)	0.019	0.23 (0.02)	0.91
Breakdancer	4903 (299)	4 (5)	568 (43)	0.007	0.43 (0.04)	0.84
CNVnator	1231 (39)	190 (6)	2537 (93)	0.102	0.71 (0.01)	1
ERDS	2292 (106)	15 (0.8)	1234 (63)	0.013	0.74 (0.02)	1

The mean for all samples (n = 36) is presented for the number, size, distance between CNVs and genome coverage. The standard deviation is given in parenthesis. For the genome coverage, all the standard deviations were smaller than 1%. For the inherited CNV rate the mean and standard deviation is across families.

### Quality metrics

To assess the tested algorithms' error rate, two metrics were used: (1) the rate of inherited CNVs defined as the number of CNVs in a family that were validated by comparing both twins and at least one parent by the total number of CNVs in the twins, and (2) the F1-score from the familial relationship classification test. Table 2 summarizes the average value of these quality metrics for the different tools. The inherited CNV rate is a good indicator of how reliable a tool is, as a high rate requires calls to be consistent in at least three individuals of the quartet (both twins and one parent). The F1-score is a good method of assessing randomness in CNV calls as non-specific ones would render unrelated pairs indistinguishable from twin-twin or child-parent pairs. In other words, this test would detect CNV calls influenced by mapping artefacts or by specific genomic regions. Such systematic bias would be present across all individuals (both related and unrelated), assuming consistency in the mapping algorithms and the sequencing process. When considering call consistency and randomness in a family, CNVnator and ERDS outperformed the other methods with higher inherited CNV rates (0.71 and 0.74, respectively) and perfect familial classification F1-Scores. CNVer suffered from a lower consistency in calling CNVs across related samples when compared to Breakdancer, as demonstrated by the inherited CNV rates (0.23 and 0.43, respectively, Table 2). The familial classification test revealed that CNVer’s call set had better delimitation between unrelated, twins and parent-child pairs than Breakdancer, suggesting that even though it suffered from a lower consistency within a family, it performed better at inferring the genetic relationship between individuals (Table 2).

### Agreement between tools

**Fig 1:**
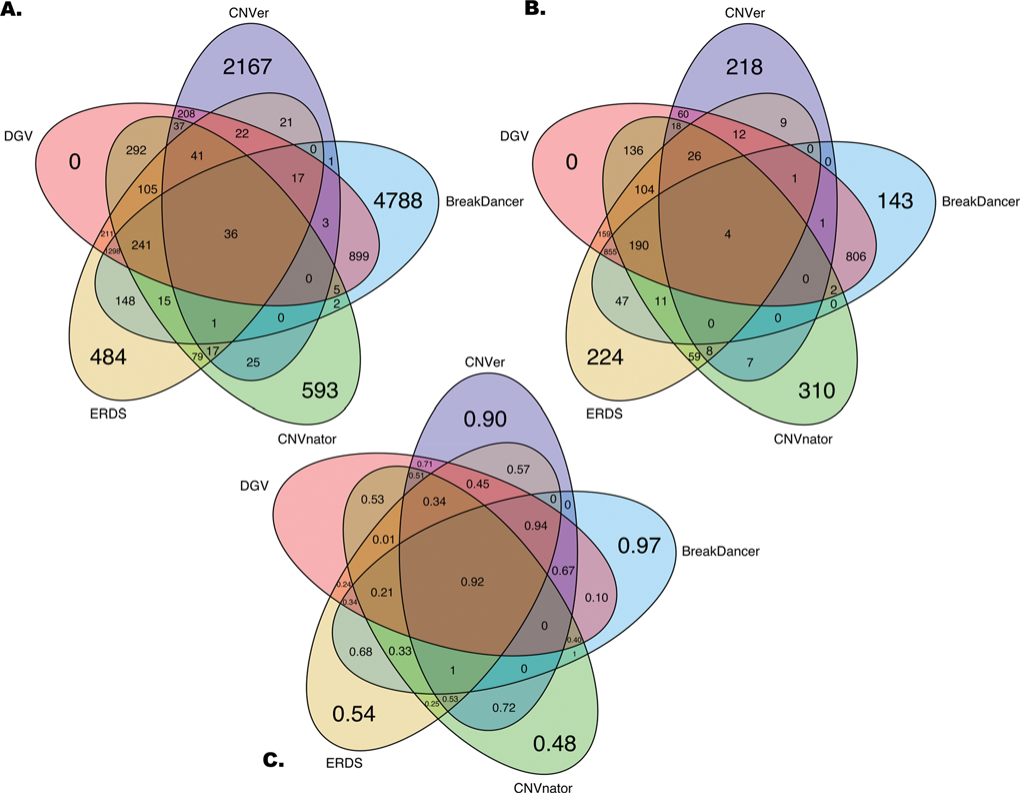
Venn diagrams representing the agreement between different CNV calling tools. The first diagram (A.) represents the mean number of CNVs shared between tools prior to any filtering based on familial relationship. B. is the mean number of CNVs shared between the tools after filtering for Mendelian inheritance (*i*.*e*. CNVs that are in both twins and at least one parent). C. is the ratio of lost CNVs when filtering for Mendelian inheritance (1-B/A). DGV is the Database of Genomic Variants.

**Fig 2:**
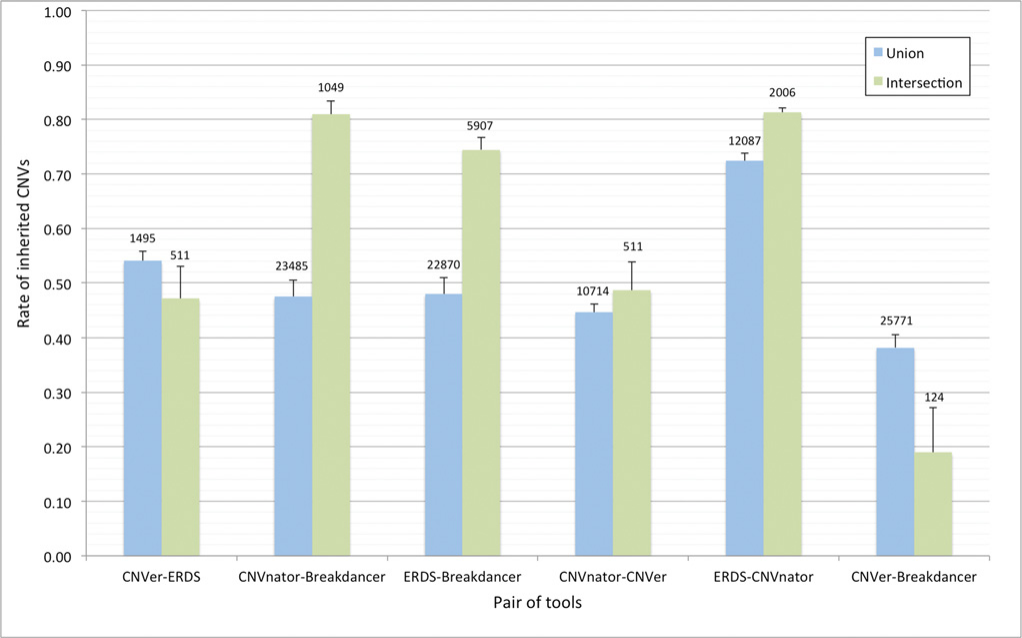
Rate of inherited CNVs when considering sets resulting from the intersection or union of pairs of tools. The rate of inherited CNVs is defined as the number of regions that were detected in both twins and at least one parent, divided by the total number of distinct regions in the twins. The union operation takes all the CNVs in either of the tools from a given pair and the intersection represents only the CNVs that were found by both tools. The mean number of CNVs in each set is provided on top of the corresponding bars.

The agreement between the tools can be visualised through the use of the Venn diagrams presented in Fig. 1. Diagrams were generated prior to any filtering (Fig. 1A) and after filtering for inherited CNVs (Fig. 1B). The difference between these two diagrams in a given space suggests a low reproducibility of the calls as filtering eliminates the CNVs that were not found in both twins and at least one parent. The reduction in the number of orphan calls for CNVer (90%) and Breakdancer (97%) is particularly striking when compared to the other tools. Such a large difference before and after filtering combined with the poor performance when considering the previously described metrics suggests that these two methods suffer from a high false positive rate. Interestingly, the set representing the CNVs that were detected using all the tools and found in the DGV database lost a high amount of calls after filtering, suggesting that the intersection of all the methods is inappropriate as this call set might be affected by sequencing or mapping biases. To find an alternative combination of methods that would be suitable for integration in genotyping pipelines, the rate of filtered CNVs for pairwise union and intersection of tools was computed (Fig. 2). The intersection between two tools being a stringent criterion, the inherited rate for intersections is generally higher than for unions, where no redundancy between CNV calls is required. However, the trade-off lies in the lower number of discovered CNVs when using the intersection between two tools. When keeping in mind the mean number of calls and the rate of inherited CNVs within a set, the intersection between ERDS and CNVnator is a suitable alternative for integration in genotyping pipelines. This solution yields, on average 2,006 CNVs where 81% were validated by the familial information. We have also compared the CNV size, GC content (of the genomic sequence) and the distance to telomeres or to the centromere between consistent and discordant CNVs in this set. A logistic regression model was used to assess the association between these sequence features and the concordance status. All of the features except the CNV size had significant association p-values after Bonferroni correction (α = 0.01/4 = 0.0025), but the effects were too small to allow efficient filtering based on these characteristics (S1 Table).

### Breakpoint detection accuracy

**Fig 3:**
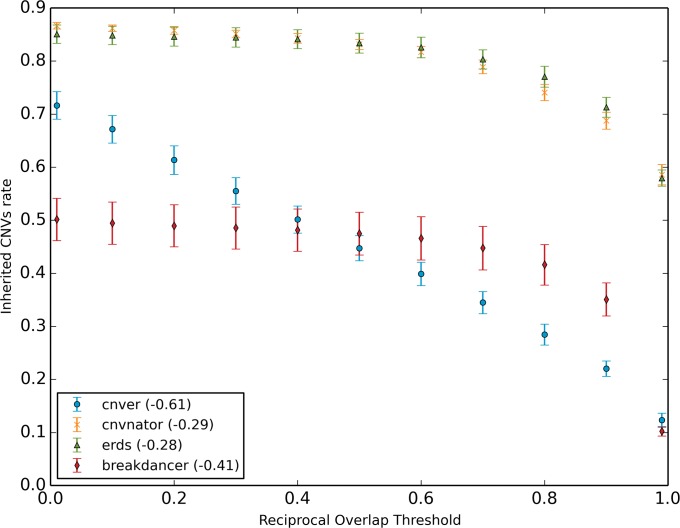
Rate of inherited CNVs as a function of the reciprocal overlap threshold used to declare copy-number variable regions identical. The slope, representing the variation in the inherited CNV rate when the reciprocal overlap threshold varies, is given in parenthesis.

One of the major limitations of previous CNV genotyping methods (*e*.*g*. microarrays or array comparative genomic hybridization) was poor breakpoint resolution resulting in CNV size overestimation and in their inability to detect smaller events [30]. This problem is expected to be less apparent in whole genome sequencing data as resolution is on the single nucleotide scale. To test the performance of the algorithms in accurately detecting CNV breakpoints, different reciprocal overlap (RO) thresholds were used to evaluate the effect on inherited CNV rate (Fig. 3). Rates varied greatly, from above 60% for small RO thresholds to under 20% for more stringent ones. To better quantify this difference, the mean slope of this curve was computed for every algorithm (Fig. 3). The negative slope for all the methods was expected as the inherited CNV rate should decrease when more rigorous thresholds are applied. Although a limited breakpoint resolution is observable for every method, CNVnator and ERDS had better performance (slopes of -0.29 and -0.27 respectively) when compared to Breakdancer and CNVer (slopes of -0.41 and -0.61 respectively). In other words, CNVnator and ERDS were less affected by the use of a more stringent overlap threshold, suggesting their breakpoint resolution is more precise.

## Conclusions

Different metrics were computed in order to thoroughly characterize the different CNV discovery methods. The use of genetically related individuals provides a valuable means of comparison as an alternative to the use of a well characterised gold standard for CNV detection. The maximum achievable inherited rate of CNVs (81%) was obtained using the intersection between ERDS and CNVnator, suggesting the importance of confirming CNV calls with independent tools. It is also noticeable that even though these two algorithms performed noticeably better than the others, roughly 20% of their calls were discordant with Mendelian inheritance and the shared genome between monozygotic twins hypothesis. This is attributable to both false positives, where a CNV is mistakenly detected in a twin, and false negatives, where a CNV is correctly identified in a twin but goes unnoticed in the identical sibling. Such discordances could partly be accounted for by using probabilistic models to integrate familial information in the discovery process [31]. Further development of tools taking into account this important genetic information, when available, would probably be greatly beneficial to the confidant genotyping of CNVs. The performance of the compared tools regarding other structural variations was beyond the scope of this article. Only CNV calls were compared, because the spectrum of detectable structural variants was variable across tools. It is also important for future studies to assess the effect of dataset specific statistics on CNV calling. The effect of depth of coverage on the efficiency of the different tools remains to be studied. Also, software related considerations, such as the importance of the parameterization of the mapping tools, needs to be further analyzed. A robust understanding of the effect of all these parameters is a crucial step towards the study of the phenotypic effect and population characteristics of CNVs.

## Supporting Information

S1 Fig Example of call fragmentation by CNVer in a family. The left histograms represent the distribution of the distance between adjacent CNVs for every sample of the family. The right pane represents the cumulative rate of CNVs separated by a given distance, *i*.*e*. the rate of CNVs affected by merging adjacent variants with a distance threshold.
(PNG)

S2 Fig Box plots of the rate of shared CNVs for different genetic relationships. Distribution of the rate of shared CNVs for 9 pairs of twins, 18 pairs of twin-parent and 9 pairs of unrelated individuals for all the considered tools.
(PNG)

S1 Table Comparison of sequence characteristics between discrepant and concordants calls for the CNVnator and ERDS intersection. Slope estimates and p-values are from a multivariate logistic regression model.
(DOCX)
