# Supplementary figures and images for: Comparison of Sequencing Based CNV Discovery Methods Using Monozygotic Twin Quartets
Source: PLoS One. 2015 Mar 26;10(3):e0122287. doi: 10.1371/journal.pone.0122287 (PMC4374778; doi:10.1371/journal.pone.0122287)

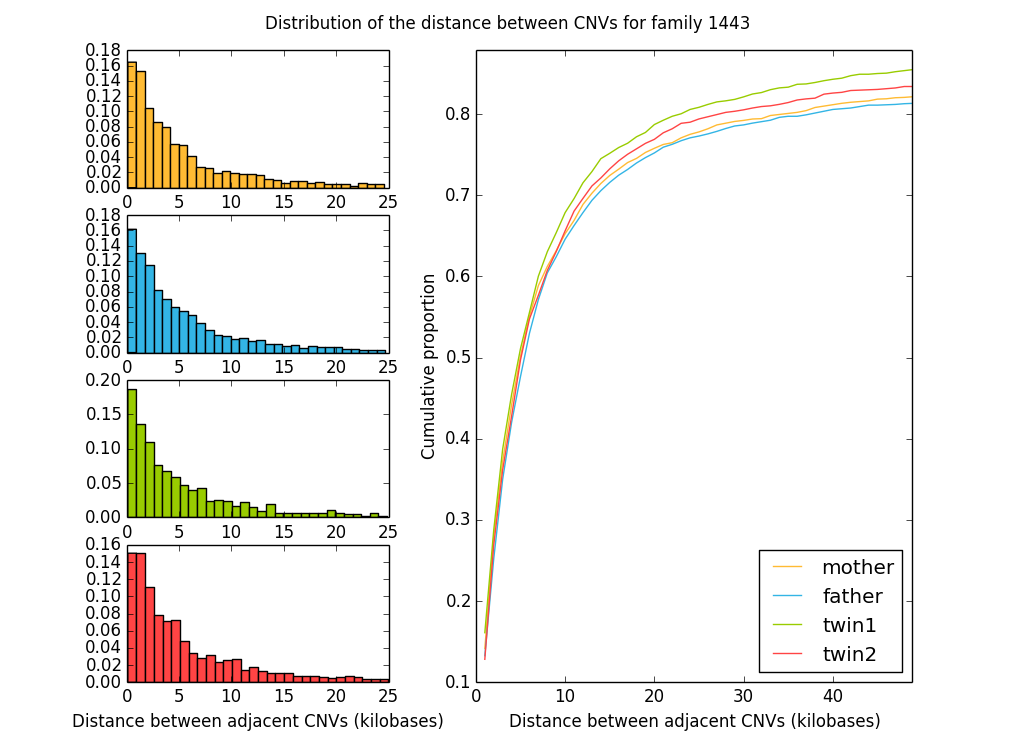

Supplement: S1 Fig — The left histograms represent the distribution of the distance between adjacent CNVs for every sample of the family. The right pane represents the cumulative rate of CNVs separated by a given distance, i.e. the rate of CNVs affected by merging adjacent variants with a distance threshold. (PNG) [file pone.0122287.s001.png]

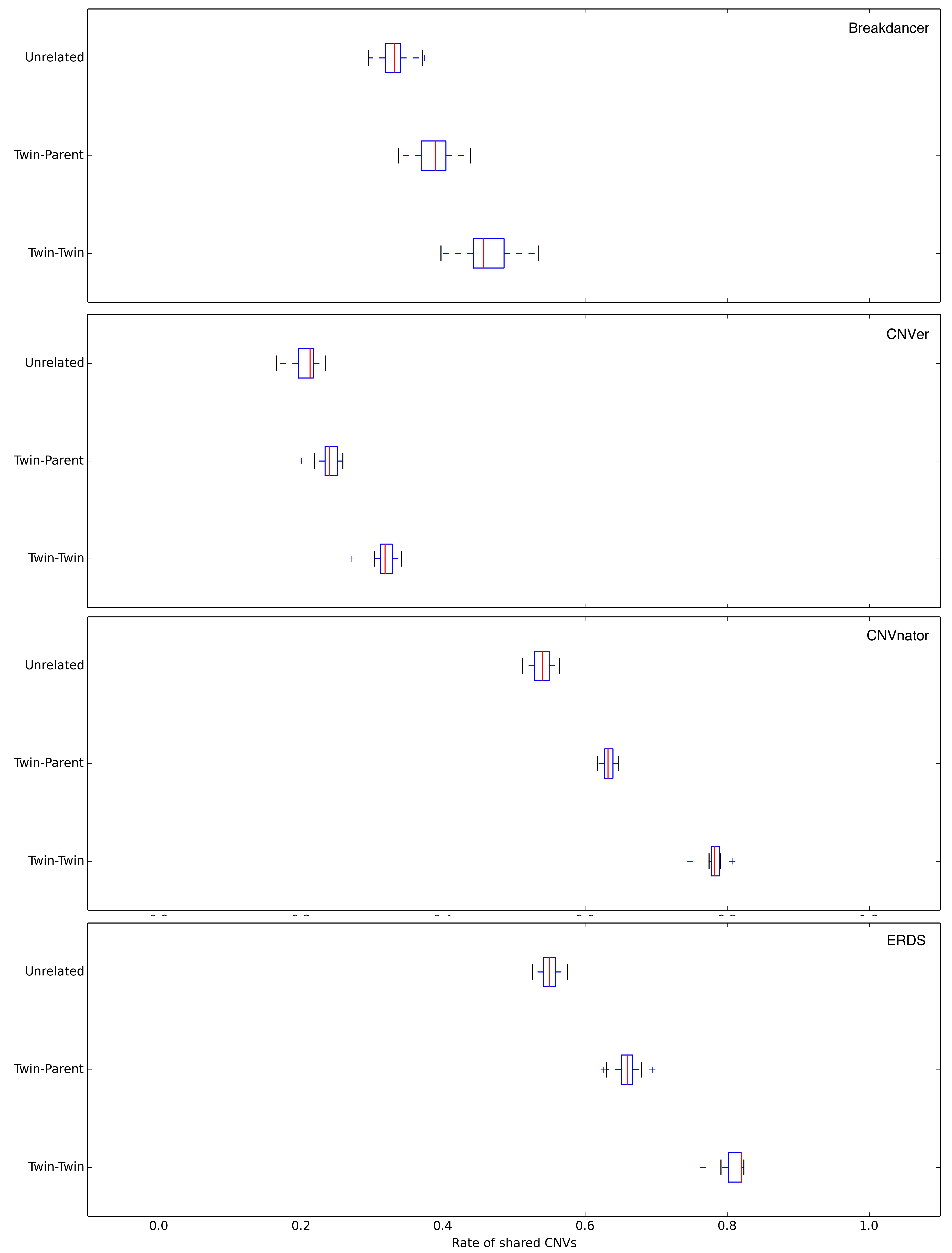

Supplement: S2 Fig — Distribution of the rate of shared CNVs for 9 pairs of twins, 18 pairs of twin-parent and 9 pairs of unrelated individuals for all the considered tools. (PNG) [file pone.0122287.s002.png]
