# Supplementary material for: Comparison of Sequencing Based CNV Discovery Methods Using Monozygotic Twin Quartets
Source: PLoS One. 2015 Mar 26;10(3):e0122287. doi: 10.1371/journal.pone.0122287 (PMC4374778; doi:10.1371/journal.pone.0122287)
Supplement: S1 Table — Slope estimates and p-values are from a multivariate logistic regression model. (DOCX) [file pone.0122287.s003.docx]

|  | Slope estimate | p-value |
| --- | --- | --- |
| Intercept | $8.89\times{10}^{-1}$ | $2.00\times{10}^{-16}$ |
| GC content | $-6.23\times{10}^{-1}$ | $1.02\times{10}^{-11}$ |
| Distance to nearest telomere | $7.71\times{10}^{-10}$ | $8.38\times{10}^{-4}$ |
| Centromere distance | $8.60\times{10}^{-10}$ | $2.19\times{10}^{-5}$ |
| CNV Size | $-1.39\times{10}^{-7}$ | $1.29\times{10}^{-1}$ |
